# Electromyographic biofeedback training for reducing muscle pain and tension on masseter and temporal muscles: A pilot study

**DOI:** 10.4317/jced.52867

**Published:** 2016-12-01

**Authors:** Laura Criado, Antonio de La Fuente, Margarita Heredia, Javier Montero, Alberto Albaladejo, José-María Criado

**Affiliations:** 1 PhD, Student. Universidad de Salamanca. Dep. Cirugía. Facultad de Medicina. Avenida Alfonso X el Sabio s/n. 37007. Salamanca. Spain; 2 PhD, MD. Associate Professor. Universidad de Salamanca. Dep. Fisiología y Farmacología. Facultad de Medicina. Avenida Alfonso X el Sabio s/n. 37007. Salamanca. Spain; 3 PhD. Senior Lecturer. Universidad de Salamanca. Dep. Fisiología y Farmacología. Facultad de Medicina. Avenida Alfonso X el Sabio s/n. 37007. Salamanca. Spain; 4 PhD. Associate Professor. Universidad de Salamanca. Dep. Cirugía. Facultad de Medicina. Avenida Alfonso X el Sabio s/n. 37007. Salamanca. Spain; 5 PhD, MD. Senior Lecturer. Universidad de Salamanca. Dep. Fisiología y Farmacología. Facultad de Medicina. Avenida Alfonso X el Sabio s/n. 37007. Salamanca. Spain

## Abstract

**Background:**

Due to the absence of agreement about an effective unified treatment for temporomandibular disorders, non-invasive therapies such as EMG-biofeedback generate a greater interest. Furthermore, most studies to the present show methodological deficiencies that must be solved in the future, which makes important to emphasize this line of studies.

**Material and Methods:**

Fourteen patients were selected for this case series study, and replied to a questionnaire concerning awareness of bruxism, painful muscles, and muscle tension. They also practiced an intraoral exploration (occlusal analysis and mandibular dynamics), and an extraoral exploration of the head and neck muscles and the temporomandibular joint. Before each session, patients responded to a questionnaire about the subjective perceived improvement. In each session, a period of three minutes of pre-biofeedback EMG activity of right masseter and temporal muscles was registered, then patients performed 30 iterations of visual EMG-biofeedback training and finally, a period of three minutes of post-EMG activity was also registered for those muscles. Patients performed four sessions.

**Results:**

A decrease in painful symptoms was found for all patients since the first session. EMG activity decreases (*p*<0,05) in both muscles during the biofeedback training stage, in the four sessions. It is also observed a decrease (*p*<0,05) in EMG activity in the masseter muscle at the post-biofeedback stage, in the second and third sessions. There is likewise a decrease in EMG post-biofeedback activity of the temporal muscle (*p*<0,05) in sessions two, three, and four.

**Conclusions:**

EMG-biofeedback training produces a decrease in EMG activity in both masseter and temporal muscles during the session. This decrease persists during the post-biofeedback period since the second session. Also there is a decrease in painful symptoms for all patients.

** Key words:**Muscle tension, muscle pain, EMG-biofeedback, masseter muscle, temporal muscle.

## Introduction

Hypotheses concerning the etiology of temporomandibular disorders still diverse and controversial (1), but there is evidence that constant clenching behaviour, even low-level contraction may lead to significant pain, soreness, tenderness or stiffness of masticatory muscles (2-4). This disorder is the cause of other common problems such as tooth wear, periodontal disease, facial pain, and headache (5).

In order to decrease muscle pain and muscle tension, different techniques have been used with unequal results (3,6-9). Surface electromyography (EMG) is an appropriate method to obtain faithful measures of frequency, intensity, and duration of muscle contraction (8,10-16). EMG-biofeedback is a self-control training of muscle activity, based on a constant feedback of EMG signal registered in a certain muscle, with the goal of modifying it. This treatment has been used in clinical and research applications and neuromuscular studies, and also in fields such as sport, neurophysiology, rehabilitation and bruxism (3,9,11,17).

The aim of this study is to evaluate the effectiveness of EMG-biofeedback training to reduce muscle tension of masseter and temporal muscles, as well as its effect on a decrease in painful symptoms, with the goal of designing a suitable protocol for a clinical context.

## Material and Methods

-Subjects

14 patients (7 men and 7 woman; mean age 22,9±4,9; median was 21 years old) were included in this study, who met the following inclusion criteria: (i) subjective awareness of awake bruxism; (ii) subjective muscle stiffness; and (iii) two or more items described below: pain around the temporomandibular joint, causing discomfort in the morning; tooth indentation on the cheek mucosa and/or tongue; masticatory muscle hypertrophy; bone torus; dental attrition of the mandibular incisors (9,18); or diagnosis of myofascial pain according to the Research Diagnostic Criteria for Temporomandibular Disorders (RDC-TMD) from the International RDC-TMD Consortium (19).

For this study, patients replied to a questionnaire containing items to determine the prevalence of bruxism and symptoms of temporomandibular dysfunction, concerning items such as awareness of bruxism, painful muscles or muscle tension according to the RDC-TMD (19). They were practiced an intraoral exploration (occlusal analysis and mandibular dynamics), and an extraoral exploration of the head and neck muscles and the temporomandibular joint according to the RDC-TMD (19). The degree of muscle tension was objectively determined by an exploration of craniofacial muscles and by recording the baseline EMG activity of right masseter and temporal muscles (20), and also subjectively using an 11-point numerical rating scale ranged 0-10, where 0 is the absence of tension and 10 is the maximal tension (18). Painful symptoms were determined by an exploration of craniofacial muscles, and also the pain degree was valued, using an identical 11-point numerical rating scale (18,19).

Exclusion criteria were: wearing a removable partial denture, lack of any occlusal supporting zone due to tooth loss in the molar region, current use of muscle relaxants or anti-inflammatory drugs, or advanced periodontal disease (9,18).

**Figure 1:**
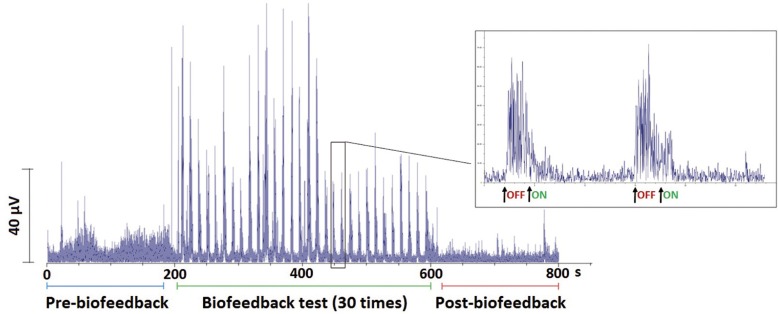
EMG-BIOFEEDBACK TRAINING PROTOCOL. The figure shows the sequence of an EMG recording of the masseter muscle during the different stages of the EMG-biofeedback protocol. On the amplified figure, we can see two of the EMG-biofeedback protocol cycles, which shows the starting and ending points of the EMG-biofeedback training cycle.

The protocol used for those patients in each session was as follows (Fig 1).

Previous to the EMG recording, patients replied to a questionnaire to determine the degree of perceived improvement after the therapy in order to find changes among sessions.

1. For a period of three minutes, baseline pre-biofeedback (preBFB) EMG activity of right masseter and temporal muscles was simultaneously recorded. To minimize EMG artifacts, patients were asked to maintain a natural erect position (21), and neither to speak nor to move their heads or bodies during the whole EMG recording;

2. Right after, they were applied the EMG-biofeedback training (BFB) with visual feedback. Patients watched a moving bar, which showed the amplitude of their muscular contraction. This training consisted of 30 iterations of a basic cycle with a ten-second-interval, during which the patient must keep the moving bar under a previously established threshold, by relaxing their masticatory muscles; and with another three-second-interval with no specific activity. The threshold was individually established in a value of a 20% lower than the patient’s baseline average EMG activity.

3. Next, for a period of three minutes, baseline post-biofeedback (postBFB) EMG activity of right masseter and temporal muscles was simultaneously recorded.

Each session was performed twice a week for two consecutive weeks.

This research was approved by the Ethics Committee of the Universidad de Salamanca. All patients signed an informed consent after they received a detailed explanation about experimental procedures, possible discomforts, and risks. All the experimental procedures were non invasive.

-Electromyogram recording

For the recording of the EMG activity, four 10-mm-diameter Ag/AgCl contact electrodes, (Lessa), were used. Electrodes were applied on the skin over right masseter and temporal muscles, separated 15 mm one from each other, and faced in the same direction of muscular fibres. One minute was waited before starting the recording, in order to reduce electric resistance. A fifth reference electrode, located far from the muscle to be explored, was also used and systematically applied over the right mastoid process, according to Ferrario *et al.* (11) (1991) and Farella *et al.* (22) (2009) and the Surface ElectroMyoGraphy for the Non-Invasive Assessment of Muscles (SENIAM) standards. In addition, for procedure standardization, both electrode placing and EMG recording where always performed by the same experimenter.

-Data analysis procedure.

The electrodes were connected to a differential amplifier (Brainclinics), model Brainquiry QPET2, 200 Hz sampling rate, 24 bits resolution. The digitalized signal was sent through bluetooth connection to a Windows 7 PC computer with software Bioexplorer version 1.6 (CyberEvolution). The obtained signal was pass-band filtered (2-100 Hz) to minimize noise and interferences. The digitizer used a notch filter (50-60 Hz) to remove electrical network interferences.

Further analysis of the registered electromyographic signal were performed by using the software Bioreview version 1.6b (Cybe-revolution). The obtained signal was smoothed by averaging one-second intervals every 500 ms to obtain a simplified representation of the signal. Thirty seconds with neither noise nor artifacts were extracted from each of the analyzed stages: preBFB, BFB, and postBFB.

Mean amplitude and integrated EMG of both masseter and temporal muscles were evaluated for each stage of the EMG-biofeedback training protocol (preBFB, BFB and postBFB). The BFB stage and the postBFB stage were compared to the initial baseline situation (preBFB stage) in all the four sessions. As well, it was evaluated the effect of the technique on patient’s symptoms, measured at the moment of their selection.

-Statistical analysis.

In order to simplify the statistical analysis of compiled data, representative groups of EMG values were extracted at preBFB and postBFB periods.

The descriptive statistic used the mean and standard deviation for quantitative variables, and the number and percentage of subjects in qualitative variables. For the EMG analysis, the intrasubject comparisons of the mean amplitude, and integrated EMG of both masseter and temporal muscles during the follow-up observations, were calculated by Paired-T-Tests with the baseline recordings as references. In the same sense, the sample distributions tabulated according to distinct qualitative variables, were compared longitudinally by using the McNemar test for related samples, with the baseline distribution as reference.

The Statistical Package for the Social Sciences v.20. (SPSS Inc., Chicago, IL) was used for the statistical analyses. The cut-off level for statistical significance was 0.05.

## Results

-Symptoms

A decrease in painful symptoms was found for all patients since the first sessions.  Table 1 shows the improvement perceived by patients, according to the symptoms they showed at the initial anamnesis.

**Table 1:**
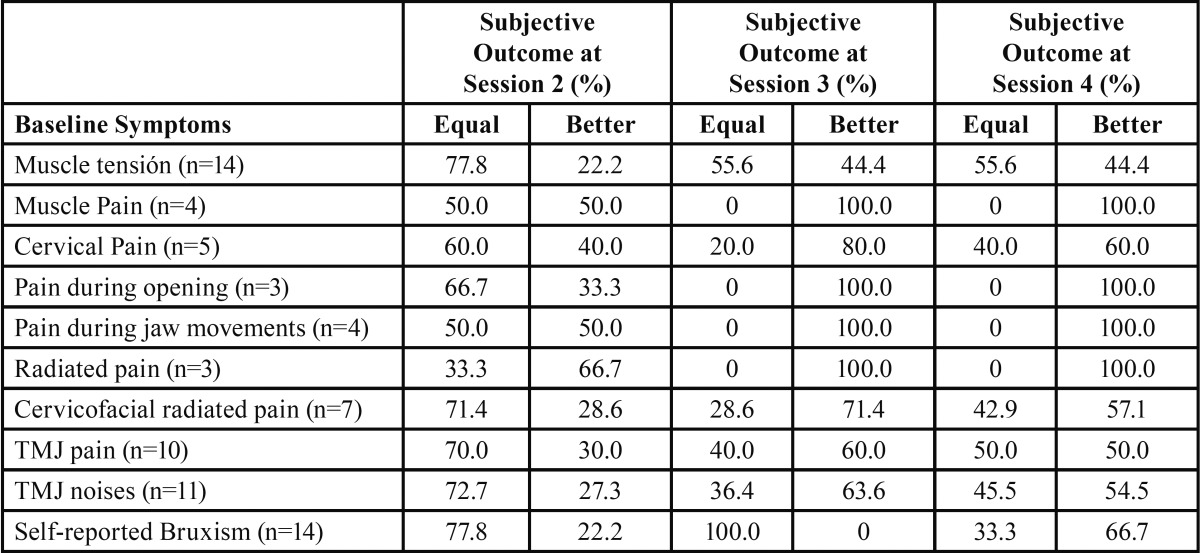
Non-parametric tests for assessing the effect of relevant symptoms on the subjective outcome during 4 sessions follow-up. McNemar tests (n=14) (%).

Comparing the second session with the first one, 50% of patients with ‘muscle pain’ as well as ‘pain during jaw movements’, showed signs of improvement. All patients with ‘muscle pain’, ‘pain at opening movements’, ‘pain during jaw movements’, and ‘radiated pain’ showed a significant subjective improvement at sessions three and four, in reference to previous sessions. 80% of patients with ‘cervical pain’ showed signs of improvement in the third session. Also other parameters, as ‘muscle tension’ (44,4%), ‘cervicofacial pain’ (71,4%), ‘TMJ noises’ (63,6%), and ‘TMJ pain’ (60%), showed improvement in the third session.

Besides, patients who showed more painful and tension symptoms before the treatment, showed a more significant improvement (*p*<0,05).

-Masseter EMG Activity.

 Table 2 shows the evolution of masseter muscle EMG activity in the above mentioned three stages analysed during the four sessions.

**Table 2:**
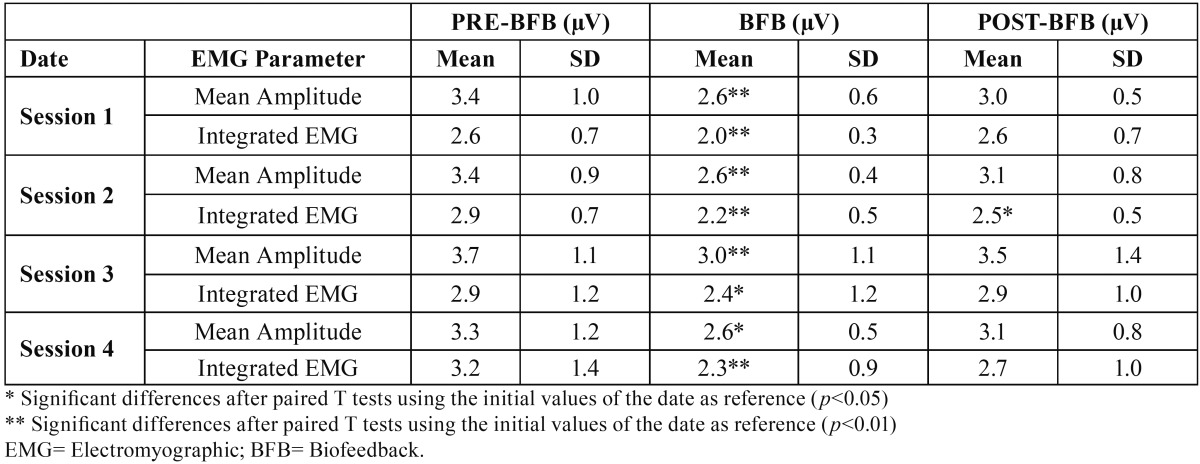
EMG assessment of the muscular parameters at the initial, experimental and final observations recorded in the right masseter during four experimental dates (n=14).

In the four sessions, it was observed during the BFB stage, a significant (*p*<0,05) or very significant (*p*<0,01) decrease in EMG activity related to the preBFB stage, for all the parameters except for the mean frequency.

When comparing the postBFB and preBFB stages, a reduction of EMG activity was found for all sessions for most of the studied variables, though only integrated EMG showed a statistically significant decrease for postBFB stage, in the second session (*p*<0,05).

-Temporal EMG Activity 

 Table 3 shows the evolution of EMG activity of temporal muscle in the three stages of the test, during the four sessions. During the BFB period, there was a decrease in EMG activity compared to the preBFB stage, which was significant (*p*<0,05) or very significant (*p*<0,01) for all the parameters.

**Table 3:**
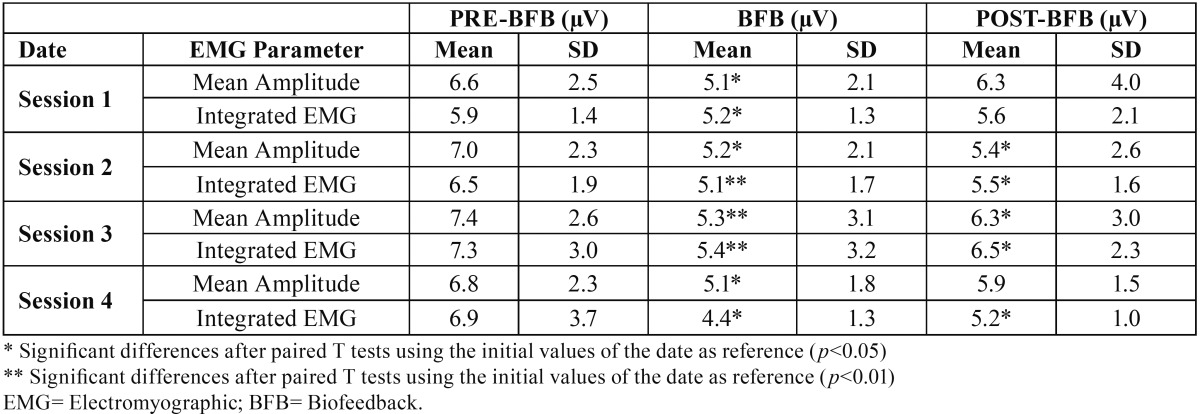
EMG assessment of the muscular parameters at the initial, experimental and final observations recorded in the right temporal during four experimental dates (n=14).

Comparing the postBFB and preBFB stages, there was a significant postBFB decrease in mean amplitude (*p*<0,05) and integrated EMG (*p*<0,05) in the second and the third session. In the fourth session, there was also a decrease in registered activity, which is significant for integrated EMG (*p*<0,05).

## Discussion

This study reveals that, both at individual level and statistical analysis of the studied population, there is a decrease in EMG amplitude, which systematically appears during the training stages with EMG-biofeedback training (BFB stage). Such decrease is statistically significant in both masseter and temporal muscles, thus explaining the immediate effect of muscle relaxation with BFB therapy.

Decrease in EMG activity during BFB stage is due to patient’s voluntary relaxation, and it reflects the correct performance of BFB test. Decrease in mean amplitude during BFB matches with other authors’ findings (13,14,18,23), and reflects a generalized reduction of muscle tension, which points out that EMG-biofeedback training may be useful to regulate an excessive muscle activity. These results support the suitability of this technique, appropriate as therapeutic method for patients with excessive muscle tension caused by clenching behaviour or other causes.

There are also differences between muscle activity registered postBFB compared to the one registered preBFB, which reflects that the patient is able to maintain the muscle relaxation obtained during the BFB stage, and involves the presence of weaker EMG events (smaller amplitude), and even, their absence. In general, there seems to be a more stable answer from the temporal muscle, whose postBFB EMG activity significantly diminishes after sessions two, three and four. Decrease in postBFB EMG activity, points out a moderate duration of the effect, especially in the temporal muscle.

BFB produces masticatory muscle relaxation, but signs of improvement cannot only depend on muscle relaxation, but also on different factors, such as personality, attitude, motivation, and psychological involvement of the patient, which also can influence on a successful treatment, as other authors express (14). Such aspects are difficult to evaluate when the studied population is reduced, so that it would be necessary to increase the sample.

It has also been observed that muscle pain (even cervical pain) decreases, and even disappears along the four sessions with BFB, which coincides with other authors’ findings (18,23,24). Decrease in EMG activity during the BFB stage is linked to patients’ physical and mental relaxation. In fact, most patients experienced a general relaxation at the end of each session, though there are evident tendencies, coherent with the EMG results and the hypothesis that says that BFB produces an improvement of symptoms.

Consequently, EMG-biofeedback training might be a useful therapeutic tool to efficiently treat symptoms in patients with occlusal pathology as clenching behaviour, temporomandibular disorders, muscle pain, soreness, tenderness or stiffness of masticatory muscles, as other authors report (18,23,24).

It has been also found that patients who show signs of improvement, also had more pain and muscle tension symptoms before starting the treatment than those who did not improve at all (*p*<0,05). This would indicate that signs of improvement are more evident for patients with more symptoms at the beginning of the treatment, compared to those with less severe symptoms. This fact can affect assessments about the success of this technique, because although there is a decrease in EMG activity, and consequently of muscle tension and activity, the patient could be not always aware of his/her own improvement.

Although this study did not find significant differences among the sessions, the reduced number of sessions perhaps makes difficult to find similar results to the work by Wieselmann-Penkner *et al.* (14) (2001), who also pointed out an improvement of the patients skills to detect, control, and reduce the muscle tension.

In spite of the study’s limitations, this work shows that EMG-BFB produces a decrease in EMG activity, for both masseter and temporal muscles, and an improvement of painful symptoms in all patients, which suggests the use of this technique as a adequate therapeutical method for reducing muscle tension. This study also suggests that EMG-BFB shows a learning effect that enables patients to identify and maintain a mandibular position where the average level of muscle activity remains low. These results encourage to do further research with long-term assessment.

The association of muscle tension with stress and anxiety levels (5,13,21,25-28), and the fact that stress is a part of our daily life makes necessary to develop therapeutic protocols for the correct management of this common clinical problem. Contemporary standards of treatment for temporomandibular disorders emphasize conservative and reversible treatments such as EMG-biofeedback. In addition, methodological deficiencies shown in most studies must be solved in the future, which makes important to emphasize this line of studies (13,15,25,29,30).

## References

[1] Carlsson GE (2009). Critical review of some dogma in Prosthodontics. J Prosthodont Res.

[2] Svensson P, Burgaard A, Scholosser S (2001). Fatigue and pain in human jaw muscles during a sustained, low-intensity clenching task. Arch Oral Biol.

[3] Fujisawa M, Kanemura K, Tanabe N, Gohdo Y, Watanabe A, Iizuka T (2013). Determination of daytime clenching events in subjects with and without self-reported clenching. J Oral Rehabil.

[4] Manfredini D, Lobbezoo F (2010). Relationship between bruxism and temporomandibular disorders: a systematic review of literature from 1998 to 2008. Oral Surg Oral Med Oral Pathol Oral Radiol Endod.

[5] Ohayon MM, Li KK, Guilleminault C (2001). Risk factors for sleep bruxism in the general population. Chest.

[6] Pierce CJ, Gale EN (1988). A comparison of different treatments for nocturnal bruxism. J Dent Res.

[7] de la Hoz-Aizpurua JL, Díaz-Alonso E, LaTouche-Arbizu R, Mesa-Jiménez J (2011). Sleep bruxism. Conceptual review and update. Med Oral Patol Oral Cir Bucal.

[8] Wang LF, Long H, Deng M, Xu H, Fang J, Fan Y (2014). Biofeedback treatment for sleep bruxism: a systematic review. Sleep Breath.

[9] Sato M, Iizuka T, Watanabe A, Iwase N, Otsuka H, Terada N (2015). Electromyogram biofeedback training for daytime clenching and its effect on sleep bruxism. J Oral Rehabil.

[10] Barbosa TdeS, Miyakoda LS, Pocztaruk RdeL, Rocha CP, Gavião MB (2008). Temporomandibular disorders and bruxism in childhood and adolescence: Review of the literature. Int J Pediatr Otorhinolaryngol.

[11] Ferrario VF, Sforza C, D'Addona A, Miani A Jr (1991). Reproducibility of electromyographic measures: a statistical analysis. J Oral Rehabil.

[12] Ardizone I, Celemin A, Aneiros F, del Rio J, Sanchez T, Moreno I (2010). Electromyographic study of activity of the masseter and anterior temporalis muscles in patients with temporomandibular joint (TMJ) dysfunction: comparison with the clinical dysfunction index. Med Oral Patol Oral Cir Bucal.

[13] Lavigne GJ, Khoury S, Abe S, Yamaguchi T, Raphael K (2008). Bruxism physiology and pathology: an overview for clinicians. J Oral Rehabil.

[14] Wieselmann-Penkner K, Janda M, Lorenzoni M, Polansky R (2001). A comparison of the muscular relaxation effect of TENS and EMG-biofeedback in patients with bruxism. J Oral Rehabil.

[15] Ilovar S, Zolger D, Castrillon E, Car J, Huckvale K (2014). Biofeedback for treatment of awake and sleep bruxism in adults: systematic review protocol. Syst Rev.

[16] Moreno I, Sánchez T, Ardizone I, Aneiros F, Celemin A (2008). Electromyographic comparisons between clenching, swallowing and chewing in jaw muscles with varying occlusal parameters. Med Oral Patol Oral Cir Bucal.

[17] Rainoldi A, Melchiorri G, Caruso I (2004). A method for positioning electrodes during surface EMG recordings in lower limb muscles. J Neurosci Methods.

[18] Watanabe A, Kanemura K, Tanabe N, Fujisawa M (2011). Effect of electromyogram biofeedback on daytime clenching behavior in subjects with masticatory muscle pain. J Prosthodont Res.

[19] Schiffman E, Ohrbach R, Truelove E, Look J, Anderson G, Goulet JP (2014). Diagnostic Criteria for Temporomandibular Disorders (DC/TMD) for Clinical and Research Applications: recommendations of the International RDC/TMD Consortium Network and Orofacial Pain Special Interest Group. J Oral Facial Pain Headache.

[20] Lobbezoo F, Ahlberg J, Glaros AG, Kato T, Koyano K, Lavigne GJ (2013). Bruxism defined and graded: an international consensus. J Oral Rehabil.

[21] Ferrario VF, Tartaglia GM, Galletta A, Grassi GP (2006). The influence of occlusion on jaw and neck muscle activity: a surface EMG study in healthy young adults. J Oral Rehabil.

[22] Farella M, Palla S, Gallo LM (2009). Time-frequency analysis of rhythmic masticatory muscle activity. Muscle Nerve.

[23] Dohrmann RJ, Laskin DM (1978). An evaluation of electromyographic biofeedback in the treatment of myofascial pain-dysfunction syndrome. J Am Dent Assoc.

[24] Crockett DJ, Foreman ME, Alden L, Blasberg B (1986). A comparison of treatment modes in the management of myofascial pain dysfunction syndrome. Biofeedback Self Regul.

[25] Vanderas AP, Menenakou M, Kouimtzis T, Papagiannoulis L (1999). Urinary catecholamine levels and bruxism in children. J Oral Rehabil.

[26] Lobbezoo F, Lavigne G (1997). Do bruxism and temporomandibular disorders have a cause-and-effect relationship?. J Orofac Pain.

[27] Kampe T, Edman G, Bader G, Tagdae T, Karlsson S (1997). Personality traits in a group of subjects with long-standing bruxing behaviour. J Oral Rehabil.

[28] Kato T, Thie NM, Huynh N, Miyawaki S, Lavigne GJ (2003). Topical review: sleep bruxism and the role of peripheral sensory influences. J Orofac Pain.

[29] Bronfort G, Haas M, Evans R, Leininger B, Triano J (2010). Effectiveness of manual therapies: the UK evidence report. Chiropr Osteopat.

[30] Crider A, Glaros AG, Gervitz RN (2005). Efficacy of Biofeedback-Based Treatments for Temporomandibular Disorders. Appl Psychophysiol Biofeedback.

